# In vitro optical quality measurements of three intraocular lens models having identical platform

**DOI:** 10.1186/s12886-017-0460-0

**Published:** 2017-06-29

**Authors:** Hyeck Soo Son, Tamer Tandogan, Stephanie Liebing, Patrick Merz, Chul Young Choi, Ramin Khoramnia, Gerd U. Auffarth

**Affiliations:** 1 David J. Apple International Laboratory for Ocular Pathology and International Vision Correction Research Centre (IVCRC), Department of Ophthalmology, University of Heidelberg, Heidelberg, Germany; 2 Department of Ophthalmology, Kangbuk Samsung Hospital, Sungkyunkwan University, Seoul, South Korea

**Keywords:** IOL, Optical quality, MTF, USAF target, Light loss

## Abstract

**Background:**

With recent advances in technology and introduction of new intraocular lens (IOL) models, surgeons today have the opportunity to choose from various optical designs, which can influence the postoperative quality of vision. In our laboratory study, we compared the optical quality of three different IOLs that use the identical platform and are produced by the same manufacturer. The study included two diffractive multifocal IOLs, a bifocal and a trifocal one, as well as a monofocal IOL.

**Methods:**

Three IOL models: monofocal CT ASPHINA 409 M, diffractive bifocal AT LISA 809 M, and diffractive trifocal AT LISA Tri 839MP (Carl Zeiss Meditec AG, Germany) were assessed for optical quality by measuring modulation transfer function (MTF) and Strehl Ratio (SR) values at pupil sizes of 3.0 and 4.5 mm on the OptiSpheric® IOL PRO (Trioptics GmbH, Germany). The United States Air Force (USAF) Target images were also recorded to comfirm the optical performance qualitatively.

**Results:**

For far focus at 50 lp/mm and 3.0 mm pupil size, MTF value of the monofocal lens (MTF = 0.798) was 1.8-fold and 2.1-fold better than the bifocal (MTF = 0.446) and the trifocal (MTF = 0.382) IOLs, respectively. For near focus, bifocal IOL (MTF = 0.265) was 1.4-fold better than trifocal IOL (MTF = 0.187), while for intermediate focus, the trifocal IOL (MTF = 0.148) was 1.7-fold better than the bifocal IOL (MTF = 0.086). For the same pupil size, total sum of light loss amounted to 5.2% for the monofocal, 16.0% for the bifocal and 6.0% for the trifocal IOL. For a larger pupil, the amount of light loss increased significantly for the multifocal IOLs.

**Conclusions:**

The monofocal IOL performed the best for far, the bifocal IOL for near and the trifocal IOL for intermediate focus. While the monofocal IOL created the least amount of light loss for both pupil sizes, the trifocal IOL created less than half the amount of light loss than the bifocal IOL for small pupil. For large pupil, however, less light scatter was observed for the bifocal than the trifocal IOL.

## Background

Cataract is considered the leading cause for blindness worldwide and impairs the vision of millions of the global population today [1]. Its treatment involves surgical extraction of the opacified crystalline lens using phacoemulsification with subsequent implantation of an intraocular lens (IOL) into the capsular bag [2]. Monofocal IOLs, which restore excellent unaided visual acuity for far vision, are currently the most prevalently implanted lenses [3]. Multifocal IOLs are gaining in popularity as they provide satisfactory unaided intermediate and near vision while maintaining good distant vision [4].

The multifocal IOLs have different strategies to achieve simultaneous vision at multiple focal points: most use a refractive or a diffractive optic design, while more recent models also feature convolution or apodization to improve contrast sensitivity and reduce the incidence of dysphotopsia [5, 6].

Numerous studies have sought to compare the optical performance of different multifocal IOL models in an effort to describe their characteristics and match their optical behavior with the requirements of individual patients [7–11]. Variations from one model to another in multifocal IOL optical quality can be related to the lens platform (the lens design and lens material) as well as to the lens optics. In the present laboratory study, we compared the optical performance of two diffractive multifocal IOLs, a bifocal and a trifocal, both of which share the design platform of a monofocal IOL produced by the same manufacturer.

## Methods

### Intraocular lenses

CT ASPHINA 409 M is a single-piece, monofocal IOL with an aspheric, aberration-neutral design. The lens is composed of hydrophilic-acrylic (25% water content) material with hydrophobic surface. It features an optic diameter of 6.0 mm on a total lens diameter of 11.0 mm and can be implanted into the capsular bag through a microincision of 1.5 mm, thereby minimizing surgically-induced astigmatism. The lens is available in a power range from 0.0 to +32.0 D. It can be acquired in 1.0 D increments for 0.0 to +10.0 D, in 0.5 D increments for +10.0 to +30.0 D, and in 1.0 D increments for +30.0 to +32.0 D.

AT LISA 809 M is a single-piece, full-diffractive bifocal IOL with +3.75 D addition for near vision. It is also composed of hydrophilic-acrylic (25% water content) material with hydrophobic surface and has an optic diameter of 6.0 mm on a total diameter of 11.0 mm, allowing implantation through a microincision of 1.5 mm. It is available in a power range from 0.0 to +32.0 D in 0.5 D increments.

AT LISA Tri 839MP is a single-piece, diffractive trifocal IOL with +3.33 D near addition and +1.66 D intermediate addition. It is also composed of hydrophilic-acrylic (25% water content) material with hydrophobic surface properties and has an optic diameter of 6.0 mm on a total diameter of 11.0 mm, opt for implantation through a microincision of 1.8 mm. Only the central area of 4.34 mm diameter functions trifocally, while the peripheral area is a bifocal optic and diffracts light rays to far and near foci. The IOL is available in a power range of 0.0 to +32.0 D in 0.5 D increments.

Five samples of each of the three IOL models were assessed – a total number of 15 IOLs. All the studied IOLs have a base power of +21.0 D.

### Optical quality assessment

Optical quality was evaluated via IOL metrology using a professional optical bench equipment (IOL OptiSpheric® Pro, Trioptics GmbH, Germany). The equipment set-up and measurement principles comply with the guidelines stated by the International Standard Organization (ISO) 11,979–2 [12] and 11,979–9 [13] and thus include an aberration-free model cornea, with which both quantitative and qualitative analyses can be performed at various spatial frequencies and apertures.

A light source radiates light rays with wavelength of 546.1 nm [14]. These are collected into parallel beams by a collimator and illuminate the target of interest. The target can be selected as a cross slit for MTF measurement or a USAF target image. The light beams then enter the test IOL which is placed in a model eye, which contains saline with refractive index of 1.336 at ambient temperature. The test IOL focuses the projected target at its focal plane, which is captured by the measurement detector including an objective microscope lens and a high-resolution charge-coupled device (CCD) camera with integrated autofocus mechanism. The captured image of the target is processed to assess the optical quality.

### Optical quality parameters

MTF and Strehl Ratio (SR) values as well as the USAF target images were studied to assess the optical quality.

MTF is a widely accepted and validated parameter [15, 16] routinely used to quantify the lens optical performance. MTF is measured by generating the line spread function (LSF) from the captured image, which reflects the ability of an optical system to reproduce an infinitesimal thin slit image [14]. The cross-sectional intensity profile of the slit image is then computed into the MTF values via Fourier-Transform technique. After measuring the MTF, IOL OptiSpheric® Pro performs a Through-Focus Scan (TFS) by moving the CCD camera along the focal planes of the optical axis and calculating all the MTF values at the spatial frequency of 50 lp/mm. In this study, the MTF values were measured at spatial frequencies of 50 and 100 lp/mm, which is equivalent to 20/40 and 20/20 Snellen visual acuity, respectively [3], at apertures of 3.0 and 4.5 mm, which represent the pupil sizes that patients over the age of 60 have under photopic and mesopic conditions, respectively [17]. Furthermore, for the purpose of this study, the MTF values of AT LISA 809 M for the intermediate focus were obtained where the lens demonstrated its highest MTF values (at approximately +1.87 D for 3.0 mm and +2.64 D for 4.5 mm aperture.

As the area under the 2-dimensional MTF curve is an absolute measure, the IOL’s optical quality can also be quantified by dividing the area below the measured MTF curve by the area below the diffraction-limited MTF curve [6, 18]. The calculated ratio is the SR, a parameter for the assessment of the IOL’s optical quality over the span of all spatial frequencies [8, 19, 20]. As SR takes into account all the small oscillations that occur on the MTF curve, it reflects the overall optical performance. A perfect IOL would have a SR of 1.0. The smaller the SR value, the worse the optical quality [18]. Then, from the measured SR values, the amount of lost light was calculated by using the equation: *Light Loss = 1 – Total SR Value*, as total light scatter is represented by the difference between the area under the diffraction-limited MTF curve and the area under the measured MTF curve.

In addition to the measurements related to the MTF values, an USAF image test was performed to qualitatively confirm the optical performance of the IOLs. Again, for AT LISA 809 M, the USAF images for the intermediate focus were recorded where the lens showed its best optical quality.

### Statistical analysis

All measured data were imported into an Excel database (Office 2010, Microsoft Corp). In order to increase the significance and reliability of the obtained results, the MTF values were measured 10-times independently for each IOL, from which a mean value was calculated. From the five mean values for each IOL model, a final mean value was calculated that served to represent the optical performance of the IOL model. An independent two-sample t-test was performed using the MedCalc statistical software for Windows (Version 15, MedCalc Software, Belgium) to assess whether the mean values of the IOL models for each parameter were significantly different from each other or not. The calculated *P*-value of ≤0.05 was considered statistically significant.

## Results

**Fig. 1:**
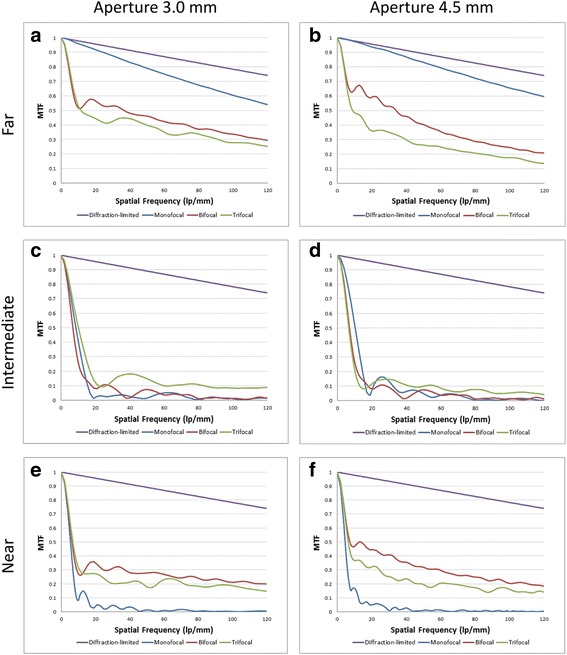
MTF curves of the IOLs for far (Sections **a**, **b**), intermediate (Sections **c**, **d**), and near foci (Sections **e**, **f**) at both apertures

Figure 1 demonstrates the MTF curves for the studied IOL at apertures of 3.0 and 4.5 mm for far, intermediate, and near focal points.

### MTF values at 50 lp/mm and 3.0 mm pupil size (Table 1)

For *far focus*, the monofocal IOL showed the highest MTF value (MTF = 0.798), which is almost twice the values of the bifocal (MTF = 0.446) or the trifocal IOL (MTF = 0.382). The trifocal IOL had the highest MTF value for the *intermediate focus* (MTF = 0.148), followed by the bifocal IOL (MTF = 0.086). For *near focus*, the bifocal IOL demonstrated superior MTF value (MTF = 0.265) than the trifocal one (MTF = 0.187). The measured MTF values of all 3 IOL models showed statistically significant difference from each other for each focus.

**Table 1:** MTF values at 50 lp/mm in 3.0 mm pupil size and statistical analyses

	MTF at 50 lp/mm in 3.0 mm Pupil Size		
	CT ASPHINA 409 M	AT LISA 809 M	AT LISA Tri 839MP
Far	**0.798**	**0.446**	**0.382**
*p-value*	to bifocal: *p < 0.0001*	to trifocal: *p < 0.0001*	to monofocal: *p < 0.0001*
Intermediate	**0.015**	**0.086**	**0.148**
*p-value*	to bifocal: *p < 0.0001*	to trifocal: *p = 0.0017*	to monofocal: *p < 0.0001*
Near	**0.018**	**0.265**	**0.187**
*p-value*	to bifocal: *p < 0.0001*	to trifocal: *p = 0.0001*	to monofocal: *p < 0.0001*

### MTF values at 50 lp/mm and 4.5 mm pupil size (Table 2)

Again, for *far focus*, the monofocal IOL showed the most dominant MTF value (MTF = 0.825). The trifocal IOL had the highest MTF value for *intermediate focus* (MTF = 0.108) and for *near focus*, the bifocal IOL (MTF = 0.311) outperformed the trifocal counterpart (MTF = 0.182). The MTF values of the monofocal (MTF = 0.051) and bifocal (MTF = 0.088) IOLs for *intermediate focus* did not show a statistically significant difference from each other (*p* = 0.0612).

**Table 2:** MTF values at 50 lp/mm in 4.5 mm pupil size and statistical analyses

	MTF at 50 lp/mm in 4.5 mm Pupil Size		
	CT ASPHINA 409 M	AT LISA 809 M	AT LISA Tri 839MP
Far	**0.825**	**0.406**	**0.265**
*p-value*	to bifocal: *p < 0.0001*	to trifocal: *p = 0.0008*	to monofocal: *p < 0.0001*
Intermediate	**0.051**	**0.088**	**0.108**
*p-value*	to bifocal: *p = 0.0612*	to trifocal: *p = 0.0008*	to monofocal: *p = 0.0038*
Near	**0.009**	**0.311**	**0.182**
*p-value*	to bifocal: *p < 0.0001*	to trifocal: *p = 0.0004*	to monofocal: *p < 0.0001*

### MTF values at 100 lp/mm and 3.0 mm pupil size (Table 3)

Also for the spatial frequency of 100 lp/mm, the MTF value of the monofocal lens for *far focus* (MTF = 0.618) was nearly double the value of the bifocal lens (MTF = 0.321). For *intermediate focus*, the peak MTF value was measured in the trifocal IOL (MTF = 0.087). The bifocal IOL outperformed the trifocal IOL for *near focus* again, but their values did not prove to have a statistically significant difference from each other (*p* = 0.0708).

**Table 3:** MTF values at 100 lp/mm in 3.0 mm pupil size and statistical analyses

	MTF at 100 lp/mm in 3.0 mm Pupil Size		
	CT ASPHINA 409 M	AT LISA 809 M	AT LISA Tri 839MP
Far	**0.618**	**0.321**	**0.259**
*p-value*	to bifocal: *p < 0.0001*	to trifocal: *p = 0.0017*	to monofocal: *p < 0.0001*
Intermediate	**0.018**	**0.011**	**0.087**
*p-value*	to bifocal: *p = 0.0111*	to trifocal: *p < 0.0001*	to monofocal: *p < 0.0001*
Near	**0.004**	**0.211**	**0.191**
*p-value*	to bifocal: *p < 0.0001*	to trifocal: *p = 0.0708*	to monofocal: *p < 0.0001*

### MTF values at 100 lp/mm and 4.5 mm pupil size (Table 4)

For *far focus*, the monofocal IOL had a MTF value (MTF = 0.651) that was more than twice the values of the bifocal (MTF = 0.244) or the trifocal IOL (MTF = 0.175). Again, the trifocal and bifocal IOLs showed peak MTF values for *intermediate* (MTF = 0.061) and *near foci* (MTF = 0.206), respectively.

**Table 4:** MTF values at 100 lp/mm in 4.5 mm pupil size and statistical analyses

	MTF at 100 lp/mm in 4.5 mm Pupil Size		
	CT ASPHINA 409 M	AT LISA 809 M	AT LISA Tri 839MP
Far	**0.651**	**0.244**	**0.175**
*p-value*	to bifocal: *p < 0.0001*	to trifocal: *p < 0.0001*	to monofocal: *p < 0.0001*
Intermediate	**0.010**	**0.011**	**0.061**
*p-value*	to bifocal: *p = 0.9052*	to trifocal: *p < 0.0001*	to monofocal: *p < 0.0001*
Near	**0.002**	**0.206**	**0.151**
*p-value*	to bifocal: *p < 0.0001*	to trifocal: *p* < 0.0001	to monofocal: *p < 0.0001*

### Strehl ratio and light loss for 3.0 mm pupil size (Table 5)

Of the 3 IOL models, the monofocal IOL showed the highest SR value (SR = 0.948), with mere 5.2% scattered light. The bifocal and trifocal IOLs showed total SR values of 0.840 and 0.940, respectively, with bifocal lens (Light Loss = 16.0%) thus having more than twice as much light loss than the trifocal lens (Light Loss = 6.0%).

**Table 5:** Comparison of Strehl Ratio and Light Loss values measured at 3.0 mm pupil size

	Strehl Ratio in 3.0 mm Pupil Size		
	CT ASPHINA 409 M	AT LISA 809 M	AT LISA Tri 839MP
Far	0.948	0.506	0.449
Intermediate			0.196
Near		0.334	0.295
Total SR	0.948	0.840	0.940
Light Loss	5.2%	16.0%	6.0%

### Strehl ratio and light loss for 4.5 mm pupil size (Table 6)

For 4.5 mm pupil size, the monofocal IOL showed the highest SR value (SR = 0.845) and the lowest amount of light lost (Light Loss = 15.5%). The bifocal IOL had higher SR value (SR = 0.625) than the trifocal IOL (SR = 0.543), which was reflected in its superior efficiency in terms of light loss (Light Loss = 37.5%) than its trifocal counterpart (Light Loss = 45.7%).

**Table 6:** Comparison of Strehl Ratio and Light Loss values measured at 4.5 mm pupil size

	Strehl Ratio in 4.5 mm Pupil Size		
	CT ASPHINA 409 M	AT LISA 809 M	AT LISA Tri 839MP
Far	0.845	0.346	0.240
Intermediate			0.111
Near		0.279	0.192
Total SR	0.845	0.625	0.543
Light Loss	15.5%	37.5%	45.7%

### USAF targets recorded for both pupil sizes (Figs. 2 and 3)

**Fig. 2:**
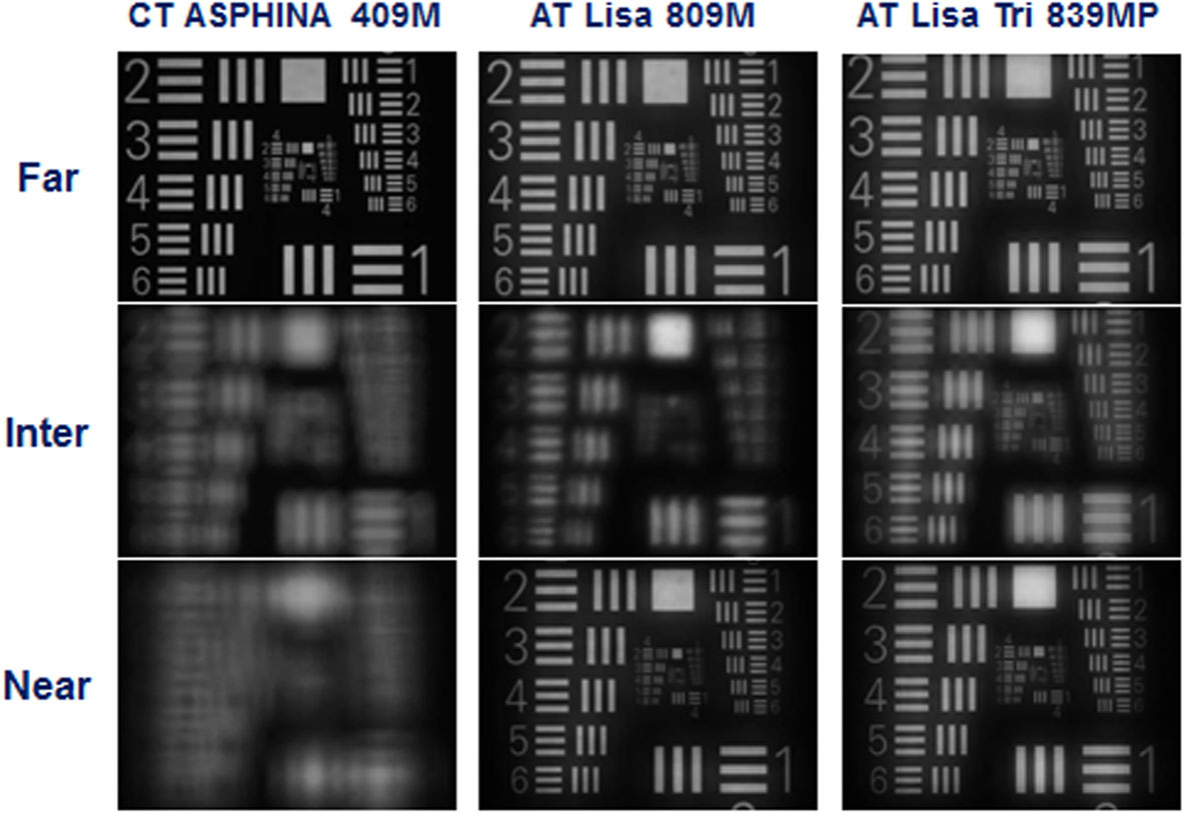
USAF target images of the 3 IOLs recorded at 3.0 mm pupil size for far, intermediate, and near focal points

**Fig. 3:**
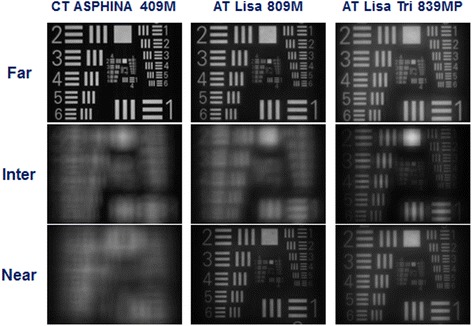
USAF target images of the 3 IOLs recorded at 4.5 mm pupil size for far, intermediate, and near focal points

As shown in Figs. 2 and 3, the recorded USAF target images for the 3 IOL models illustrated results that are comparable to those of measured MTF and SR values, with the monofocal IOL showing the best optical quality for *far focus*, the bifocal IOL for *near focus* and the trifocal IOL for *intermediate focus*.

### Through-focus MTF scan for both pupil sizes (Fig. 4)

**Fig. 4:**
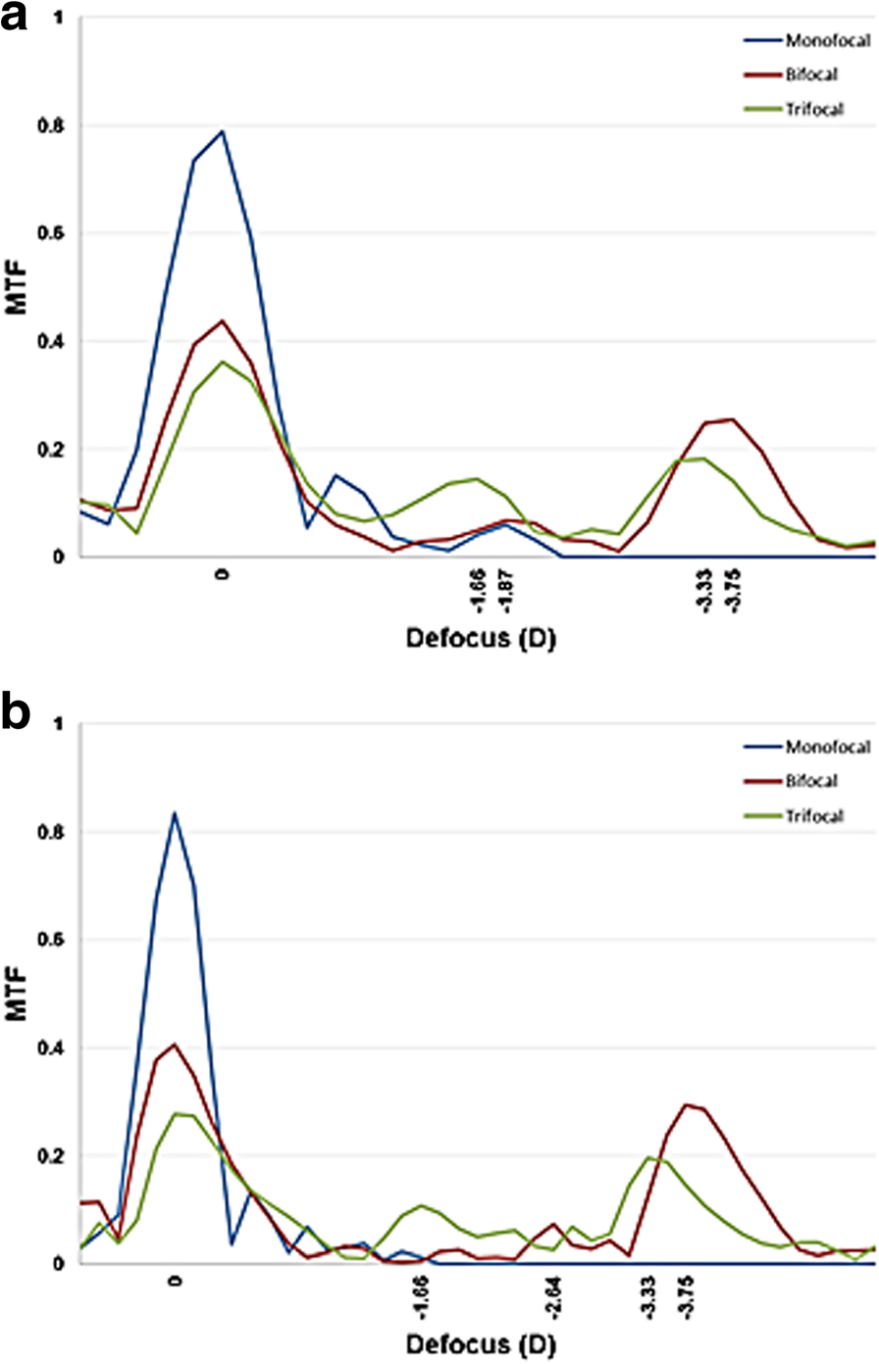
Through-Focus Scan of the 3 IOLs measured at 3.0 mm (**a**) and 4.5 mm (**b**) pupil sizes. The MTF values were calculated at spatial frequency of 50 lp/mm

The through-focus MTF curves of the three IOLs at spatial frequency of 50 lp/mm are shown in Fig. 4 for pupil sizes 3.0 mm (A) and 4.5 mm (B). For both pupil sizes, the monofocal IOL yielded 1 peak for *far focus*, while the bifocal and trifocal IOLs demonstrated 3 peaks corresponding to *far, intermediate, and near foci*. The defocus diopter values for the two multifocal IOLs at their peak *intermediate* and *near foci* were noted. At 3.0 mm aperture, the monofocal IOL had the highest MTF peak for *far focus*. The trifocal IOL had higher MTF peak for *intermediate focus* at −1.66 D than the bifocal IOL at approximately −1.87 D, while for *near focus*, the bifocal IOL demonstrated higher MTF peak at −3.75 D than the trifocal IOL at −3.33 D. Similarly, at 4.5 mm aperture, the monofocal IOL outperformed the two multifocal IOLs for *far focus*, while the bifocal and trifocal IOL showed dominant results for *near* (bifocal IOL at −3.75 D and trifocal IOL at −3.33D) and *intermediate* (bifocal IOL at −2.64 D and trifocal IOL at −1.66 D) *foci*, respectively.

## Discussion

The design and technology of IOLs have undergone numerous modifications in recent years. Monofocal IOLs can restore excellent vision for far focus, yet require patients to always have spectacles at hand to compensate for the missing foci. Multifocal IOLs provide more depth of focus and functional vision in more than one focal point that potentially allows independence from spectacles [4] and an increase in quality of life [21]. Among the different types of multifocal IOLs that are available, one can differentiate those that employ a pseudo-accommodating design with a refractive, a diffractive, or a combined diffractive-refractive optical profile, from those that employ an accommodating one.

Numerous trials attempted to analyze and compare the optical quality of various multifocal IOLs. It has been shown that IOLs exhibit differing optical characteristics depending on the material and the optical profile they use [7]. In a laboratory study, Artigas et al. compared two different bifocal diffractive IOLs and observed that AcrySof ReSTOR SN60D3 performed superior for distant vision at all pupil apertures than Tecnis ZM900, which performed better for near vision [8]. The authors attributed such difference in the optical behavior to the slight variance in the intended goal of the two diffractive IOLs: AcrySof ReSTOR SN60D3 is designed to create near vision while simultaneously maintaining satisfactory image quality at far distance, whereas Tecnis ZM900 attempts to improve near visual acuity at the expense of far visual acuity. It is also important to note that both IOLs possess different materials: ReSTOR uses acrylic material, while ZM900 is based on silicone. Other studies have also reported heterogeneity in the optical performance of various diffractive bifocal IOLs produced by different manufacturers [9–11].

The optical behavior of the two trifocal diffractive IOLs: Finevision Micro F (PhysIOL, Liege, Belgium) and AT LISA Tri 839MP have also been extensively studied. Carson et al. reported that in 3.0 mm pupil size and 50 lp/mm, the two trifocal lenses showed comparable MTF values for far (MTF = 0.342 and MTF = 0.330 for AT LISA 839MP and FineVision, respectively), intermediate (MTF = 0.136 and MTF = 0.140), and near (MTF = 0.213 and MTF = 0.230) focal points [3]. In a larger pupil, however, Ruiz-Alcocer et al. observed that FineVision performed better for far focus, while AT LISA Tri 839MP outperformed FineVision for intermediate and near foci [22]. Such differing optical performances are ascribable to the variance in the IOL platforms they employ. Although both IOLs are aspheric and composed of hydrophilic acrylic materials, AT LISA Tri 839MP features an additional hydrophobic surface and the two IOLs differ in the principal structure of their optical profiles and distribution of light [23, 24]. Furthermore, FineVision additionally features convoluted steps and an apodized diffractive optical surface that renders this IOL distance-dominant with increasing pupil size.

Evidently, IOLs of different manufacturers show varying optical behavior as each manufacturer uses an IOL platform that is not fully identical in lens design, material, and profile as that of another manufacturer. In this study, we compared the optical performance of two diffractive multifocal IOLs, a bifocal and a trifocal one, both of which are based on the same IOL platform of a monofocal IOL made by the same manufacturer, in order to reduce to a minimum the impact which the lens platform can have on the optical quality.

In Through-Focus Scan, the monofocal CT ASPHINA 409 M showed one peak at its base power dedicated to far focus. Both AT LISA 809 M and AT LISA Tri 839MP showed three peaks that correspond to their base power, intermediate add, and near add. While AT LISA 809 M had a higher MTF value for near focus, AT LISA Tri 839MP had a higher MTF peak for intermediate focus. The measured MTF and SR values were also in accordance with the results of the TFS, with CT ASPHINA performing the best for far, AT LISA 809 M for near and AT LISA 839MP for intermediate focus.

With respect to light distribution, for 3.0 mm pupil, CT ASPHINA 409 M allocated 94.8% of its incident light rays to the far focus, AT LISA 809 M dedicated 50.6% to far and 33.4% to near focus, and AT LISA Tri 839MP 44.9% to far, 19.6% to intermediate and 29.5% to near focus. As a result, the sum of scattered light amounted to 5.2% for CT ASPHINA 409 M, 16.0% for AT LISA 809 M and 6.0% for AT LISA Tri 839MP. For 4.5 mm pupil, the two diffractive multifocal IOLs generated more light scatter, with AT LISA 809 M and AT LISA Tri 839MP having light loss of up to 37.5% and 45.7%, respectively. CT ASPHINA 409 M, in distinction, generated only 15.5% light scatter compared to its multifocal successors.

The recorded USAF images also confirmed the pattern of the MTF and SR results qualitatively. The image quality of CT ASPHINA 409 M showed a progressive attenuation from far to near foci at both apertures, while AT LISA 809 M and AT LISA Tri 839MP had the best image quality for near and intermediate foci, respectively. The optical performance of AT LISA 809 M was better for intermediate focus than that of CT ASPHINA 409 M, yet AT LISA Tri 839MP still had the best image quality at intermediate focus.

When aiming to compare the optical quality of IOLs, it is important that the lenses share the same IOL platform. In other words, the studied IOLs should not only be equal in base power, but also in their material and lens design.

The IOL’s material has been shown to influence the optical quality by causing longitudinal chromatic aberration (CA) [25–27]. An Abbe number reflects the amount of CA produced by the optical material and is calculated by taking into account the material’s refractive indices at different wavelengths. Contemporary IOL materials possess Abbe numbers that range from 35 to 60 [26] and those with Abbe number of 47 have been shown to produce similar amount of longitudinal CA to that of a physiological human eye [25]. In an experimental study, Zhao et al. measured the Abbe numbers of different acrylic and silicone IOLs to evaluate the influence of the CA on the IOL’s image quality and observed that IOLs of acrylic material with higher Abbe number provided superior optical performance [25]. All of the three IOLs analyzed in this study, which are composed of hydrophilic-acrylic material with hydrophobic surface, share the same Abbe number of 56.5.

The optical design of an IOL also affects the image quality. The integration of an aspheric optic profile has been found to significantly reduce the deteriorating effects of the spherical aberration (SA), thereby improving the optical performance [28]. The performance of multifocal IOLs also varies depending on their design. When a comparison is made between refractive and diffractive multifocal IOLs, studies show that the diffractive IOLs are better in terms of near visual acuity, contrast sensitivity, and dysphotopsia [29–31]. Furthermore, as there are differences in the optical design of diffractive IOLs, such as those additionally featuring apodization or convolution as an effort to improve the contrast sensitivity [5, 6], each diffractive IOL has an optical characteristic unique to its innate design.

Our results are limited to the extent that we cannot predict the optical characteristics of the 3 IOL models if the measured lenses would have a substantial difference in their base power values. In order to standardize the base power values of the studied IOLs, we determined to use +21.0 D for all the lenses, which represents the clinical average base power of these IOLs. The dioptric power for near addition also varies slightly between AT LISA 809 M and AT LISA Tri 839MP (the former has a near add at +3.75 D and the latter at +3.33 D). For the purpose of this study, the MTF values and USAF images of AT LISA 809 M for intermediate focus were measured where the lens had its best MTF value and optical quality (at approximately +1.87 D for 3.0 mm and +2.64 D for 4.5 mm pupil). Furthermore, as the model eye we used has an aberration-free cornea, it neglects the potentially adverse effects which the individual patients’ spherical aberration would have on the IOL’s optical quality in vivo. As each individual has a different ocular condition and a varying inclination for neuroadaptation that also influences the final optical quality, our results merely describe the IOL optical performance in vitro.

## Conclusion

To the authors’ knowledge, this is the first study to perform a pure comparison of optical quality in three different IOLs that share the same IOL platform. When comparing the average MTF values for far focus at 50 lp/mm and 3.0 mm pupil size, the monofocal IOL (MTF = 0.798) was 1.8-fold and 2.1-fold better than the bifocal (MTF = 0.446) and trifocal (MTF = 0.382) IOLs, respectively. For near focus, the bifocal IOL (MTF = 0.265) was 14.7-fold and 1.4-fold better than the monofocal (MTF = 0.018) and trifocal (MTF = 0.187) IOLs, while for intermediate focus, the trifocal IOL (MTF = 0.148) was 9.9-fold and 1.7-fold superior than the monofocal (0.015) and bifocal (0.086) IOLs. For far focus at 50 lp/mm and 4.5 mm pupil size, the monofocal IOL (0.825) was 2.0-fold and 3.1-fold better than the bifocal (0.406) and trifocal (0.265) IOLs, respectively. For near focus, the bifocal IOL (0.311) was 34.6-fold and 1.7-fold better than the monofocal (0.009) and trifocal (0.182) IOLs, while for intermediate focus, the trifocal IOL (0.108) was 2.1-fold and 1.2-fold superior than the monofocal (0.051) and bifocal (0.088) IOLs.

Today, IOL designs are available with different benefits and limitations. Surgeons can therefore choose the appropriate IOL platform according to each and every patient’s demands and life style. Further in vitro studies evaluating the effects of lens decentration or tilt on the IOL’s image quality may help to predict the optical behavior in clinical settings.

## Abbreviations

CA: Chromatic aberration
CCD: Charge-coupled device
IOL: Intraocular lens
ISO: International Standard Organization
LSF: Line spread function
MTF: Modulation transfer function
SA: Spherical aberration
SR: Strehl ratio
TFS: Through focus scan
USAF target: Untied States Air Force target
